# Health equity-oriented design for dissemination: products and impact of rapid community translation for COVID-19 vaccine promotion

**DOI:** 10.3389/fpubh.2026.1721808

**Published:** 2026-06-05

**Authors:** Sarah E. Brewer, Mary E. Fisher, Linda Zittleman, Amanda Skenadore, Rebecca Mullen, Emma Gilchrist, Jameel Mallory, Meredith P. Fort, Mahad Darar, Farduus Y. Ahmed, Meredith K. Warman, Jenna E. Reno, Montelle Tamez, Donald E. Nease, Bethany M. Kwan

**Affiliations:** 1 Adult and Child Center for Outcomes Research and Delivery Science, University of Colorado Anschutz Medical Campus, Aurora, CO, United States; 2 Department of Family Medicine, University of Colorado Anschutz Medical Campus, Aurora, CO, United States; 3 Colorado Clinical and Translational Sciences Institute, University of Colorado Anschutz Medical Campus, Aurora, CO, United States; 4 Office of Access and Engagement, University of Colorado Anschutz Medical Campus, Aurora, CO, United States; 5 Colorado School of Public Health, Aurora, CO, United States; 6 Colorado State University, Fort Collins, CO, United States; 7 Department of Psychiatry, University of Colorado Anschutz Medical Campus, Aurora, United States; 8 Jenna Reno Consulting LLC, Denver, CO, United States; 9 Department of Emergency Medicine, University of Colorado Anschutz Medical Campus, Aurora, CO, United States

**Keywords:** community engagement, community translation, COVID-19, dissemination, public health messaging

## Abstract

**Background:**

Public health messaging often falls short of the needs of underserved and minority communities, missing the mark on translating information that captures cultural, linguistic, or community relevance to influence health behaviors. Rapid community translation (rapid-CT) is an adaptation of the Boot Camp Translational method for community engagement in translating medical evidence into community health promotion messages and interventions. This paper describes the dissemination of materials from a rapid-CT of messages promoting COVID-19 vaccination among children and adults, boosters, and Long COVID messages.

**Methods:**

This project engaged five disproportionately impacted Colorado communities: urban and rural Latino/a/x, urban Black/African American, rural African immigrant, and urban American Indian/Alaska Native communities to conduct three cycles of about 6 week each of rapid-CT over 2021–2022. Each cycle of rapid-CT was led by 2 trained facilitators, usually one academic and one community partner. The process involved: (1) determining the role of partners and fostering partnerships; (2) describing the innovation, rationale, and evidence; (3) identifying the intended audience, message, timing, and format for dissemination; (4) selecting the communication and distribution channels; (5) identifying barriers and facilitators to dissemination, and (6) evaluating and refining the dissemination process. Dissemination was examined via tracking spreadsheet and impact was examined through surveys (recollection of seeing the materials, attitudes about COVID vaccinations, and vaccine status) and team de-briefing.

**Results:**

We engaged 126 unique community members and 15 facilitators. Within each cycle, rapid-CT communities co-created distinctive campaigns including messages, materials, and dissemination strategies. Each rapid-CT group identified anticipated and actual barriers and facilitators to the dissemination of messages and materials. Each group also demonstrated impact with metrics ranging from views of online videos or marketing materials to distributions of items promoting the message (e.g., 1,000 stickers distributed), although capacity to assess impact was a varied and the changing evidence and related to COVID-19 vaccination presented challenges.

**Conclusion:**

Rapid-CT was effective for engaging communities in co-creating distinct messages and communication strategies tailored to their community's needs and populations. Rapid-CT is well-suited to message creation for dynamic public health emergencies. Future use of Rapid-CT should set clear expectations for time commitment of community partner and establish prospective evaluation plans in addition to community-developed plans.

## Introduction

As of December 2020, vaccinations against COVID-19 were recommended for nearly all individuals in the United States, with annual boosters made available starting in September 2022 (1–5). Yet, even before COVID-19 vaccines were made widely available in early 2021, vaccine hesitancy was high (6–8). A 2020 national assessment of COVID-19 vaccination attitudes found reluctance about the vaccine was highest among Black/African American, Hispanic, and rural communities with 29–34% of each group expressing hesitancy (9). Given early COVID-19 vaccine uptake was low, (10) promotion was a public health priority in 2021 and 2022. Vaccination rates were especially low in certain communities disproportionately impacted by the effects of COVID-19, and thus special efforts were needed to engage community in designing strategies for vaccine promotion. Notably, there were greater disparities in Black and Latino/a/x communities and in rural areas. A national assessment of COVID-19 vaccination attitudes found reluctance about the vaccine was highest among Black/African American, Latino/a/x, and rural communities with 29–34% of each group expressing hesitancy (9). In April 2021, national vaccination rates for a 2-dose primary series among Black and African Americans were 46.3% and 47.3% among Hispanic/Latinx, compared to 59% among white adults (11). Uptake in rural areas was similarly low, with 46.4% of Black and 42.0% of Latino/a/x adults vaccinated against COVID-19, while 63.4% of urban white adults had received at least one dose (12).

To address these critical health disparities, a community-engaged approach to dissemination of evidence using community-specific messaging for addressing COVID-19 vaccination hesitancy was identified as high priority. A systematic review of strategies for reducing vaccine hesitancy and increasing uptake indicates that “dialogue-based interventions” that use community-focused messaging are one of the most effective in addressing vaccine hesitancy (13). The use of cultural tailoring also increases the persuasive effectiveness of health messages by increasing relevance and engagement among audience members (14, 15). Cultural tailoring strategies include designing messages that communicate relevance and a sense of familiarity, such as incorporating health data that are specific to the group, adapting content to cultural norms and values, and addressing culturally specific health facilitators and barriers (16–19).

Use of methods for community engagement and cultural tailoring for designing COVID-19 vaccine promotion dissemination strategies was the goal of the Colorado Community Engagement Alliance (CO-CEAL). CO-CEAL was funded in 2021 as one of 21 sites in the National Institutes of Health's Community Engagement Alliance (CEAL), a landmark effort to address disparities in the impacts of the COVID-19 pandemic (20–22). (https://ceal.nih.gov/who-we-are) CO-CEAL used an adapted version of Boot Camp Translation (BCT), a well-established method for community engagement in translating medical evidence into community health promotion messages and interventions.

Developed in the High Plains Research Network and its Community Advisory Council in eastern Colorado, BCT has been employed nationally and internationally in over 50 individual projects with diverse community partners and addressed numerous health topics (23–29). The BCT process results in multi-component and multi-level interventions tailored to the local community culture (30–36). BCT is effective because it fosters implementation of evidence-based practices by creating and supporting partnerships between academic researchers and local communities, increases relevance of evidence-based interventions by using language and constructs that resonate locally, and delivers messages through multi-level community channels. Since 2016, BCT has been used to address vaccine hesitancy and vaccination disparities, with projects addressing HPV vaccination in rural, suburban, and urban communities and adolescent vaccination in rural communities (37, 38). However, the traditional BCT approach takes approximately 8–10 months to complete–and given the urgency of COVID-19 vaccine promotion in 2021, a more rapid approach was needed. Furthermore, given travel restrictions and distancing requirements of the COVID-19 pandemic, there was a need to adapt BCT to be conducted in virtual spaces. For CO-CEAL, we used an adapted version of BCT, leveraging a rapid BCT process previously developed for creating community-relevant messages in urban Detroit, (39) and virtual technologies that enjoyed widespread uptake during the pandemic. The resulting rapid, virtual BCT approach–here called rapid Community Translation (rapid-CT) can be completed using virtual techniques in weeks, rather than months (40). Here, we describe and evaluate the use of the rapid-CT process, products, and dissemination to promote COVID-19 vaccination in five disproportionately-impacted Colorado communities.

We previously reported on CO-CEAL communities' transcreation process and the messages and materials developed through rapid-CT (40). In that work we described commonalities as well as unique messaging components and audiences for each community in the CO-CEAL project. Additionally, we demonstrated the feasibility of the approach to transcreate urgent public health messaging for communities experiencing disparities in a public health emergency. We found that each of the five communities translated evidence on COVID-19 vaccines in ways that emphasized the safety and effectiveness of the vaccines and the importance of vaccination against COVID-19 to protect communities as well as individuals. Each community incorporated unique cultural and community factors into their messaging and intended distribution methods. In the current paper, we focus on how materials created in the rapid-CT process were packaged and delivered to intended audiences, and how dissemination and impact were monitored and evaluated across 3 cycles of rapid-CT from 2021–2022. The methods and results are presented in the context of a 6-step dissemination planning framework that aligns with procedures for applying rapid-CT to co-designing dissemination strategies in partnership with diverse communities.

## Materials and methods

### Design and setting

CO-CEAL used the rapid-CT method to design dissemination strategies to enhance community uptake of COVID-19 vaccination. This project engaged five Colorado communities in dissemination planning and enactment: urban and rural Latino/a/x, urban Black/African American, rural African immigrant, and urban American Indian/Alaska Native [AI/AN] communities to conduct a total of 15 rapid-CT cycles in fall 2021 (cycle 1), spring 2022 (cycle 2), and fall 2022 (cycle 3). Cycle 1 focused on adult and adolescent (ages 12–17) COVID-19 vaccination, cycle 2 focused on promotion of pediatric (ages 5–17) COVID-19 vaccination, and cycle 3 incorporated updated data from the first two cycles, plus included COVID vaccine boosters, how to reach “late adopters,” and Long COVID. The facilitation teams received a truncated version of BCT facilitator training at the outset of the project on the rapid-CT methods, including an overview of dissemination theories and frameworks. Each of the five rapid-CT teams created a dissemination plan for each cycle which included intended audiences, messages, materials and packaging, communication channels (including messengers and key distribution partners), and tools for tracking and evaluating the dissemination process.

### A framework to describe dissemination planning

**Figure 1:**
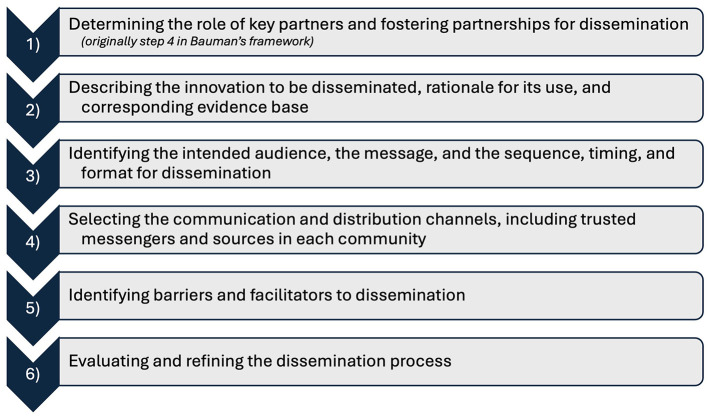
Modified framework for dissemination, adapted from bauman.

The process followed in each community is aligned with Bauman and colleagues' six-step dissemination framework (41, 42). We use Bauman's framework to describe the dissemination planning and evaluation in the CO-CEAL communities. Recognizing the importance of beginning with partnership building and community engagement, the order of the framework steps is slightly re-ordered, as follows: (1) determining the role of key partners and fostering partnerships for dissemination (originally step 4 in Bauman's framework); (2) describing the innovation to be disseminated, rationale for its use, and corresponding evidence base; (3) identifying the intended audience, the message, and the sequence, timing, and format for dissemination; (4) selecting the communication and distribution channels, including trusted messengers and sources in each community; (5) identifying barriers and facilitators to dissemination, and (6) evaluating and refining the dissemination process (Figure 1). While Bauman's framework notes the importance of fostering partnerships later in the process, our teams emphasized this from the outset and often the centrality of partnerships and community relationships and priorities. In this project, COVID-19 vaccination was considered the innovation to be disseminated. We applied Step 6 in two ways: both evaluating and refining the dissemination planning process (i.e., rapid-CT) and the dissemination plans (i.e., the products of rapid-CT and their implementation).

### Community-academic partnerships

First steps in the community group development included identifying partners (both community and academic), determining partner roles and responsibilities, and preparing teams to fulfill these roles. CO-CEAL University research faculty were the rapid-CT training and capacity building team, logistics and administration support, and process evaluation team for the work. Three faculty with extensive experience utilizing BCT with community partners led this team, including one who co-developed the original BCT process and one who co-developed the adapted version of rapid-CT. Each rapid-CT partnership team included a Community Connector, a co-facilitation pair, one of whom were members of or had a working history with the focus community and another who had experience in BCT, rapid-CT, or similar group facilitation, along with eight to fourteen community members who received a stipend for participation ($400). Community Connectors–a CO-CEAL contracted role–are community members who are trusted in the community and help recruit community partners, support data collection, and follow-up to foster engagement with the process and facilitate trust between university and community-based teams. Community Connectors work closely with the rapid-CT facilitation teams. To expand on the team roles: community members were recruited intentionally to encompass diverse community and vaccination perspectives, with an emphasis on involving parents, grandparents, and caregivers in cycle 2 and an intentional mix of people who were and were not vaccine-hesitant in cycle 3. Each team included a content expert with medical training as well as a media designer with experience creating materials for and/or with ties to the focus community (25, 26, 29, 37–39).

### Rapid-CT process

**Figure 2:**
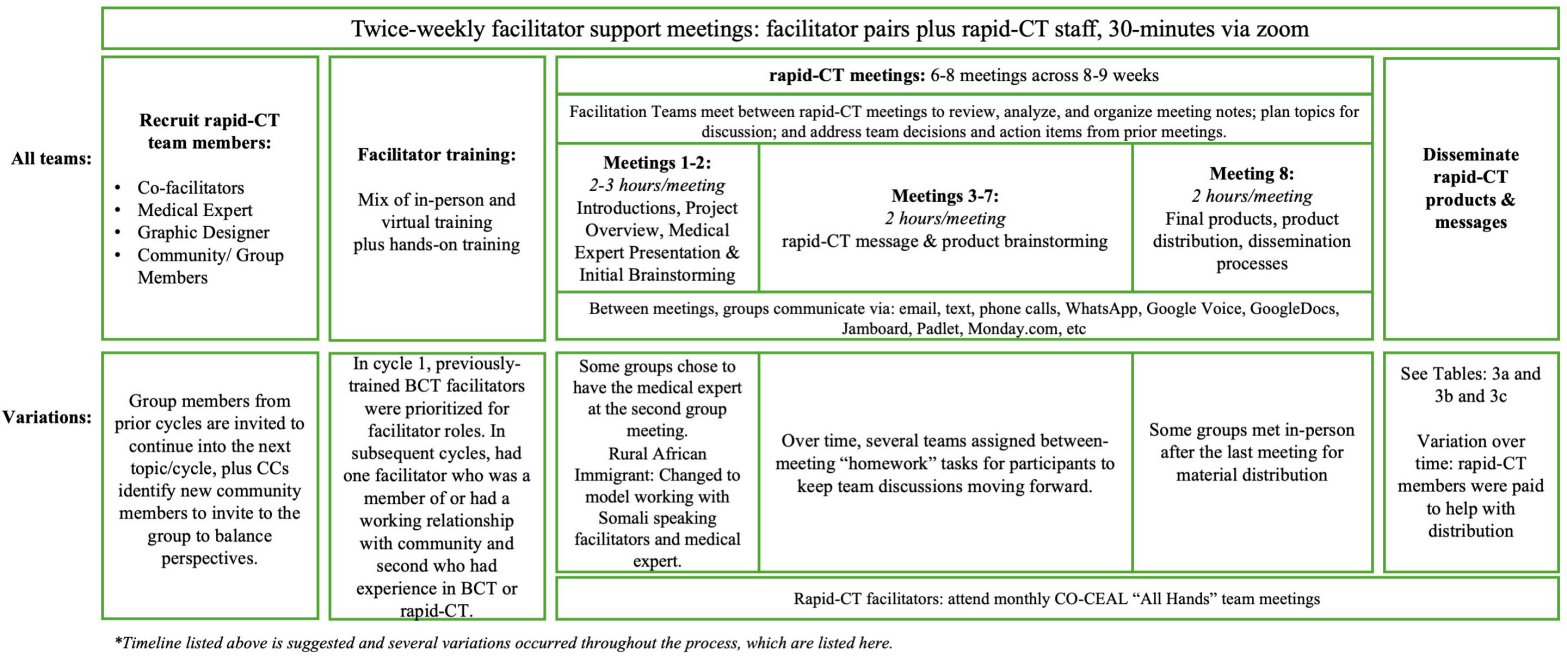
Rapid CT process and variations.

Rapid-CT operationalizes steps 2 through 6 of our adapted version of Bauman et al.'s framework (Figure 2). Rapid-CT teams met virtually (via Zoom) each week for eight to nine weeks from August to December of 2021 (cycle 1), February to June of 2022 (cycle 2), and October 2022 to February 2023 (cycle 3). In addition to group relationship building, meetings for each cycle included an educational, scientific presentation given by a medical expert on COVID-19 incidence, epidemiology, and vaccines. Each group received the same core educational content (i.e., the description, rationale, and evidence for COVID-19 vaccination) along with community-specific statistics and other cultural and historical contextual information. Subsequent meetings focused on identifying the medical evidence relevant to each community to be translated, who in the community needed to receive that information (the intended audience(s)), what message(s) would convey that information, the format and packaging of the messages, and distribution of the messages and materials to the community (to include how, when, where, and who).

Across the five focus communities, 120 community meetings and over 250 h of meeting time occurred. Most teams met for 2 h weekly by Zoom, except for an expanded 3-h “kick off” meeting for the educational presentation and discussion. Four teams were conducted primarily in English; one was conducted during cycle 1 in English with simultaneous Somali interpretation and later transitioned to be conducted primarily in Somali due to the community group understanding and learning best in their first language. This group later determined facilitation was best done with Somali-speaking facilitators and content experts only and transitioned to doing so in cycles 2 and 3.

Teams used a combination of methods to communicate during and in between rapid-CT sessions, including email, text, phone calls, WhatsApp for texting and voice messages, Google Voice for text messages, GoogleDocs, Jamboard, Padlet, and Monday.com, based on group preferences. Teams learned together and discussed group-specific topics such as the history and creation of vaccines, western vs traditional medicine, vaccine hesitancy, timeline for vaccinations, misinformation about vaccine safety, vaccine coverage for their unique community, nuances in families and intergenerational dynamics and intergenerational trauma, historical and medical mistrust, who the trusted community leaders and organizations are for each community, and Long COVID information and symptoms. Rapid-CT facilitators noted meetings included rich conversation, laughter, empathy, storytelling, discussing differences of opinion, and community capacity building.

Rapid-CT facilitators met between team meetings to review, analyze, and organize meeting notes; plan topics for discussion; and address team decisions and action items from prior meetings. As needed, and varying by each team's needs, facilitators met with Community Connectors to maintain engagement, discuss attendance and nuances of community needs, and plan for conduct of the rapid-CT work. Several teams assigned between-meeting “homework” tasks for participants to keep team discussions moving forward. Example tasks included reviewing presentation notes for important facts and asking family and friends to describe their concerns or questions about COVID-19 vaccines, as well as working on dissemination strategies such as obtaining feedback on messages and design drafts from community members, drafting scripts for a planned video, and researching public transportation advertising options.

Twice-weekly 30-min facilitator support meetings were held virtually with the rapid-CT faculty to troubleshoot challenges, share progress and facilitation or resource tips, and create a community of support. One example of how these meetings influenced facilitators' work is that early in cycle 1, facilitators from one team changed the proposed order of meetings and held the medical expert presentation at their second rather than first meeting allowing more relationship building and discussion of group norms at the first meeting. In cycles 2 and 3, most teams adopted this order. Additionally, facilitators were encouraged to attend monthly CO-CEAL “All Hands” team meetings where all project team members came together to share updates and project progress.

Facilitators documented meeting and field notes, attendance logs, facilitators' meeting summaries, draft and final campaign plans, and distribution plans. Facilitators participated in debrief meetings with rapid-CT faculty at the end of each cycle to discuss what went well and what was challenging per cycle. These documents and related discussions with the rapid-CT leadership team offered insight into the processes of each team and informed the evaluation of the effectiveness of rapid-CTs and their products. We used a modified rapid qualitative approach to summarize process data and arrive at key themes about the rapid-CT process and resultant campaigns (43, 44).

Several changes to the “traditional” rapid-CT process were suggested throughout these three cycles, including the change to hosting the medical expert at the second group meeting as shared above, as well as trying a hybrid meeting approach, of meetings done virtually mixed in with specific meetings in-person for creating or finalizing products (e.g., filming a video for the Urban Black/African American community) or finalizing dissemination plans (for the rural Latino, urban American Indian/Alaska Native, and urban Black/African American communities).

### Rapid-CT process–modification:

In cycle 1, the urban Latino community had an entire region of the community unable to fully participate in the project partway through the rapid-CT. The region that remained were able to finalize products for their specific community, but not the region that dropped out. To address the specific needs of the region that dropped out, work continued later with a new small group from that region to adapt previously-completed materials. One rapid-CT faculty member met with the CO-CEAL Community Engagement lead and five community partners to review final materials and make necessary edits prior to finalizing a dissemination plan in that specific area.

### Dissemination tracking and evaluation

Step 6 of the dissemination planning framework is to evaluate the dissemination strategies, both in terms of the process (e.g., to what extent were dissemination strategies reaching the intended audience) and impact (e.g., to what extent was there evidence that dissemination strategies were influencing COVID-19 vaccination attitudes and behavior in each community). We used several methods to attempt to track dissemination process and impact of the rapid-CT products in each of the relevant communities. The research team worked in partnership with the Community Connectors and rapid-CT teams to determine feasible and acceptable strategies for documenting distribution of products. The first iteration was a structured long-form dissemination tracker spreadsheet. The facilitation teams were first trained and asked to fill in this spreadsheet on a weekly or biweekly basis as materials were produced and distributed. At facilitator meetings, we walked through the spreadsheet and definitions of each variable. It was difficult for the facilitators to do this in a consistent way across teams (e.g., making generic to all teams vs tailored to each team); teams shared it was cumbersome and time consuming; and teams needed frequent reminders of definitions and there was confusion about terminology (e.g., “products”). They first tried to do this on their own asynchronously, with mixed results; a second strategy was to have a research team member fill it out on their behalf during synchronous meetings. This also produced mixed results. A next approach was for the research team to mine other process data, removing the burden from dissemination tracking from the facilitator teams. We also explored a simplified survey-based “drop off tracker” focused on those actively participating in the distribution of materials. This simplified survey was to be filled out by those involved in dissemination to contact facilitation teams to fill in their information manually or by self-completing an online Qualtrics survey for two communities.

Another important step was to determine how impact would be assessed, e.g., changes in attitudes and vaccination rates in the communities of interest. It was not feasible to rigorously assess unique impact on vaccination rates using population-level data–notably given the many parallel COVID-19 vaccination public health campaigns underway in the state of Colorado at the time. National CEAL program Common Surveys administered to communities at baseline and after each cycle were modified to include items about recollections of exposure to the materials, along with the standard questions regarding attitudes, beliefs, and norms about COVID-19 vaccination, trust in health information sources, current COVID-19 primary and secondary prevention behaviors, and vaccination hesitancy, plans, and current status (both flu and COVID-19). Our senior dissemination scientist met with each rapid-CT team for a debriefing after cycle 2 to discuss survey findings and community-specific ideas about what else impact would mean for their community; detailed debrief notes were taken. We then held a group discussion at a monthly CO-CEAL All-Hands meeting to assess whether there were opportunities across rapid-CT teams and communities to better understand dissemination and impact of CO-CEAL rapid-CT efforts.

At the end of each of the three cycles, the rapid-CT leadership team held 1-h debrief meetings via Zoom with each team to learn more about how the process went and suggestions for improvement, discuss the impact that they have seen within their communities, talk about future dissemination planning, and discuss next steps with the project. Those required to attend were the co-facilitation team, the community connector/s, the rapid-CT leadership, and dissemination lead, and invitations were extended to the CO-CEAL Multi-PIs and Community Engagement leads. These debriefs typically occurred within a month after the end of the rapid-CT meetings. Notes were typed up and saved from each debrief and have been analyzed for purposes of this paper. Debrief questions changed as we progressed through the three cycles to best-accommodate the changing needs of facilitation teams and the current state of the community within the COVID context. For example, in cycle 2 we asked additional questions about distribution plans to help account for previously-mentioned tracking issues and improve tracking processes; during cycle 3 debriefs we shared Boot Camp Translation material exposure results from a recent CO-CEAL community survey and sought reactions to exposure results (45) (forthcoming). The CO-CEAL dissemination lead joined these debriefs to discuss evaluating impact and decision making.

## Results

### Characteristics of engaged communities and partners (Step 1)

Across three cycles of rapid-CT in five Colorado communities, we engaged 126 unique community members and 15 facilitators (10 facilitators per cycle; 9 facilitated two cycles, 2 facilitated all 3) between August 2021 and February 2023. Descriptions of the rapid-CT teams and the community roles and demographics of team members are displayed in Table 1. Additionally, we tracked and then adapted plans, process, materials based on COVID-19 context in Table 2.

**Table 1:** Characteristics of rCT team members by community and cycle, Colorado Fall 2021–Fall 2022.

Characteristic	Rural African Immigrant	Rural Hispanic/Latino (Latinx)	Urban American Indian/Alaska Native	Urban Black/African American	Urban Hispanic/Latino (Latinx)
	Cycle 1 (Adult/Teen Vaccines)				
	*N* = 10	*N* = 16	*N* = 13	*N* = 9	*N* = 7
Male	7	6	0	2	3
Female	3	10	13	6	4
Education and Professions	Small business owners, elders, meat processing facility employees, essential workers, students	School district and early childhood care, personal care, emergency response, retired, community members, businesspeople	Native–serving organization employees, government and community–based organizations, and connections to schools and local businesses.	Health and wellness project manager, COVID vaccine clinic volunteer, business owner, philanthropist, neighborhood block captain, community activist. High School–Graduate School. Community Health and social services Professionals, Business Owners and Retirees	Small business owners, laborer, COVID-19 patient screener, life and career support, medical laboratory worker, students
Group description	Somali community members from two larger rural towns, community or spiritual leaders, community elders, business women and men and essential workers. Many knew each other from local mosques or community classes.	“Everyday” people; diversity in heritage and time in America and SLV; mix of men, women, parents, non–parents. 3 languages spoken: English, Spanish and Q'anjob'al (a Mayan dialect).	Native–led work group: designer, medical expert, community connector, one co–facilitator, workgroup participants, and the owner of the printing company identify as American Indian and represent diverse Tribes.	Mix of parents and non–parents, mostly women.	Mix of unvaccinated and those who lost loved ones to COVID; many that are Denver natives for multiple generations or have immigrated to the US.
Media Designer	Resident of same geographic rural community (not part of the community itself); experience with rapid BCT.	Resident of a (different) rural community; experience with rapid BCT.	Enrolled member of Pueblo of the Laguna (Village of Paraje) tribe; new to BCT.	Extensive experience with BCT; experience designing materials for multi–cultural communities.	Familiarity with Latinx communities; extensive experience with BCT.
Medical Expert	English–speaking physician from large FQHC with experience serving refugees (had a real–time interpreter).	CBPR–trained Latino family medicine doctor.	Adult and child medicine expert; tribal member of the American Indian pediatrician, Muscogee (Creek) Nation of Oklahoma; Associate Professor with the CSPH.	Colorado–born, medical interest in health disparities and diversity issues Extensive medical experience, a local community member, health care educator and activist.	Mexican–trained doctor, public health professional at local human services organization.
Cycle 2 (Child/Teen Vaccines)					
	*N* = 15	*N* = 17	*N* = 12	*N* = 8	*N* = 15 (7 Denver, 8 Pueblo)
Male	5	6	0	3	4
Female	10	11	11	5	11
Education and Professions	Small business owners, elders, meat processing facility employees, essential workers, students	Childcare worker, students (high school and college), school district employee	Group members are actively involved in or work for Native–serving organizations, government and community–based organizations, and have connections to schools and local businesses.	Medical and mental health professionals, childcare provider, public health and government agency staff, activists, high school–graduate school, Community Health and social services Professionals, Retirees	Caregiver, teacher, small business owner, community activists, grad student, parents and grandparents
Description of group members	Somali community members from two larger rural towns, most parents, community or spiritual leaders, community elders, businesswomen and men and essential workers. Many knew each other from local mosques or community classes.	Mix of non–parents/grandparents and parents/grandparents; Representation from all areas of SLV and different Latino cultures; Every county in the SLV except one (out of 6) had a participant for this cycle of BCT. There were more parents participating this cycle as well. Had grassroots leaders and those who needed nudging.	Majority of workgroup members continued from Cycle 1; Native–led workgroup. Community members, Elder–In–Residence, mothers, health educators, reporter/writer, healthcare professionals, and community organizations representatives and friends; They were wide age range (25–80).	Mix of parents, grandparents raising grandchildren, other grandparents; some supportive of vaccines, 3 members others hesitant.	Mix of parents and grandparents, many greatly involved in community work, teaching, and mentoring. Participants are a mix of locals, part of families who have been in CO for generations, and immigrants.
Parents	14 (93%)	at least 3	7	4 parents; 2 grandparents; 1 young adult	5 parents, 1 grandparent (5 unknown)
Media designer	Resident of same geographic rural community (not part of the community itself); experience with rapid BCT. Continued from Cycle 1.	Resident of a (different) rural community; experience with rapid BCT. Continued from Cycle 1.	Enrolled member of Pueblo of the Laguna (Village of Paraje) tribe. Continued from Cycle 1.	Extensive experience with BCT process; Excellent communicator and connector; experience designing materials for multi–cultural communities. Continued from Cycle 1.	Denver–based contemporary fine artist, illustrator, and comic book artist.
Medical expert	Somali speaking pediatrician from Washington state with experience as a refugee.	CBPR–trained Latino family medicine doctor. Continued from Cycle 1.	Adult and child medicine expert; tribal member of the Muscogee (Creek) Nation of Oklahoma. Continued from Cycle 1.	Colorado native, medical interest in health disparities and diversity issues Extensive medical experience, a local community member, health care educator and activist. Continued from Cycle 1.	Colorado–born, medical interest in health disparities and diversity issues.
**Cycle 3 (COVID-19 Vaccination and Long-COVID Prevention)**					
Male	15 members	6	0	15 members	13 members
Female		9	10	1 male/14 female	2 male/11 female
Education and Professions	Small business owners, elders, meat processing facility employees, essential workers, students	Mix of retired, Community Health Workers, Public Health, Human Resources, education/childcare, Department of Labor	Group members are actively involved in or work for Native–serving organizations, government and community–based organizations, and have connections to schools and local businesses.	Parents and grandparents, local business owner, wellness and fitness professionals, faith–based leaders, Alzheimer's association, academic staff.	Variety of professions
Description of group members	Several participated in either cycles 1 and/or 2.	There was distribution from across the 6–county region, and the group was similar to the population distributions. Additionally, 8 people had children under age 18.	All members of the group had participated in either cycles 1 and/or 2.	The majority of the group had participated in either cycles 1 and/or 2.	Comprised of Latinas/os from two major cities, had a limited number returning from past cycles, so mostly new attendees. Group was fully bilingual, which proved an advantage.
Media designer	Resident of same geographic rural community (not part of the community itself); experience with rapid BCT. Continued from Cycles 1 and 2.	Resident of a (different) rural community; experience with rapid BCT. Continued from Cycles 1 and 2.	Enrolled member of Pueblo of the Laguna (Village of Paraje) tribe. Continued from Cycles 1 and 2.	Extensive experience with BCT process; Excellent communicator and connector; experience designing materials for multi–cultural communities. Continued from Cycles 1 and 2.	Denver–based contemporary fine artist, illustrator, and comic book artist. Continued from Cycle 2.
Medical expert	Somali speaking pediatrician from Washington state with experience as a refugee. Continued from Cycle 2.	Bilingual medical doctor with training in Mexico, co–Investigator on project.	Primary care internist (Diné) with experience working with the Indian Health Service and community clinics. Originally from a small community in New Mexico on the Navajo Reservation.	Colorado native, medical interest in health disparities and diversity issues Extensive medical experience, a local community member, health care educator and activist. Continued from Cycles 1 and 2.	Colorado–born, medical interest in health disparities and diversity issues.

**Table 2:** COVID context and contemporary evidence base by CO-CEAL Cycle.

Cycle number and information	Range of dates	What was happening with COVID and evidence around vaccines	How the messages and materials required adaptations during and between cycles
Cycle 1: COVID-19 Vaccines, for Adults and Youth 16+	August–November 2021	**March–November 2020–** Clinical trials ongoing. **April 2020–** Mask orders go into effect. **December 2020–** Emergency Use Authorization (EUA); public still questioning and/or unsure of safety. **April 2021–** Johnson & Johnson vaccine paused due to blood clots; clouded the safety of all available COVID vaccines. **August 2021**– HHS, CDC, and FDA share booster vaccines will be needed for all COVID-19 vaccines; start in **September 2021** for immunocompromised and those age 65+; **November 2021–** recommended that all who received a COVID-19 vaccination get a booster.	Changes in vaccine recommendations (both brand and population) spurred revisions to messaging and broadened target audiences across all groups
**Cycle 1 Debrief Synthesis:** This was a new process for nearly all rapid–CT facilitators, and the speed at which COVID-19 information and vaccination recommendations changed was incredibly quick. Because of this, there was a lot of iteration and adaptation needed to stay current and get messages and materials with accurate information together and out into community quickly. COVID delays affected the speed at which physical materials could be printed and there was some frustration with not getting information out quickly enough. Overall, groups adapted well and materials that went into community were well–received and there was interest in continuing the work moving forward.			
Cycle 2: COVID−19 Vaccines, for children 12–15	February–April 2022	**May 2021–** Pfizer available for youth. **February 2022**–ACIP recommends the use of Moderna's vaccine for all people ages 18 years and older. **May 2022**– ACIP recommends Pfizer–BioNTech's COVID−19 vaccine boosters for everyone ages 5–11 years ACIP also recommends everyone ages 12 years and older who is immunocompromised and those ages 50 years and older should receive a second booster dose.	Increased availability for younger populations increased target audience for messaging across nearly all groups; Recommendation of boosters increased target audience for C1 messages still in circulation to those who had received initial vaccination.
**Cycle 2 Debrief Synthesis:** A shared strength was the development of trust and ownership—facilitated by culturally aligned leaders, language accessibility, and space for honest dialogue. COVID−19 vaccination recommendations continued to change around this time for both youth and adults, so the meeting frequency was able to aid in getting accurate information out. While dissemination commitments were strong across groups, some encountered challenges in finalizing detailed dissemination plans and production logistics within the short timeline. Coordination between facilitators, community co–leads, and project leadership varied, revealing a need for clearer role definitions and regular communication. Overall, the process surfaced both enthusiasm and strain—highlighting the promise of rapid engagement on this important and ever–changing topic while underscoring the resource and planning demands it places on all involved.			
Cycle 3: Long COVID, COVID−19 Boosters, “Late Adopters”	October 2022–January 2023	**Spring 2020–** An official term to describe the journeys of those not recovering (“Long COVID”). **October 2021**– WHO publishes a clinical case definition of “post COVID−19 condition” or long COVID. **June 2022**– ACIP rec ommends Moderna and Pfizer–BioNTech's COVID−19 vaccines for everyone ages 6 months−17 years **May 2023**– End of national and public health emergencies for COVID−19	End of public health emergencies increased need for messaging to address the impact on communities, including reduced funding for COVID−19 and the continued need for vaccination and other precautions based on local context.
**Cycle 3 Debrief Synthesis:** All groups deeply engaged with evolving medical information and adapted messages to reflect current realities, with a focus on accessibility and trust. Across groups, themes like long COVID, updated booster guidance, and protecting both youth and older adults gained traction, although the depth of discussion varied by context. Participants emphasized the importance of messages maintaining accurate and relevant facts. Dissemination strategies improved in intentionality, with groups exploring innovative and community–rooted methods for distribution and tracking, though many voiced a desire for simpler and more qualitative ways to assess impact. Across the board, trust, relationship–building, and community–led adaptations were essential in shaping both the content and the collaborative process.			

### Dissemination plans

The dissemination plans for each community are summarized in Tables 3a–3c, including components from dissemination planning steps 2 through 4. Details on the transcreation process and messages developed are reported elsewhere (40).

**Table 3a:** Cycle 1–Campaigns Promoting Adult and Teen COVID−19 Vaccination, Colorado, Fall 2021–Spring 2022.

rCT Team	Key messages	Description of campaign	Distribution strategies	Example product
**Rural African Immigrant**	•Community education about COVID−19 symptoms and treatment. •Empowering the community to participate in COVID−19 prevention, education and vaccination. •COVID−19 vaccine options and benefits •Personal protection measures related to COVID−19. •Resources provision. •Addressing Misinformation and Myths: Discuss common misconceptions and myths about vaccines and provide accurate information.	•Somali and English language flyers and posters, as well as in–person community presentations	•Meetings and events for community members, canvasing local businesses and restaurants to hang flyers, sharing in breakrooms at work and the meat processing facilities. •One one–on–one educational and resource sessions as culture culture–appropriate or needed	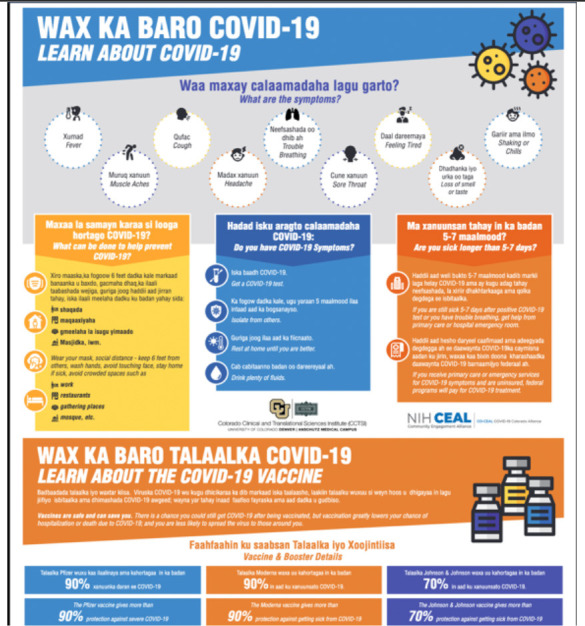
**Rural Latino/a/x**	•Read my story and start the conversation •Unvaccinated people are at higher risk for death and disease	•Spanish and English language rack cards, banners, yard signs, website, and Tik Tok competition •Messages combine stories of people who were personally affected by COVID with local and national data	•Dia de los Muertos celebration •Banners in schools and other local businesses and gathering places •Public event	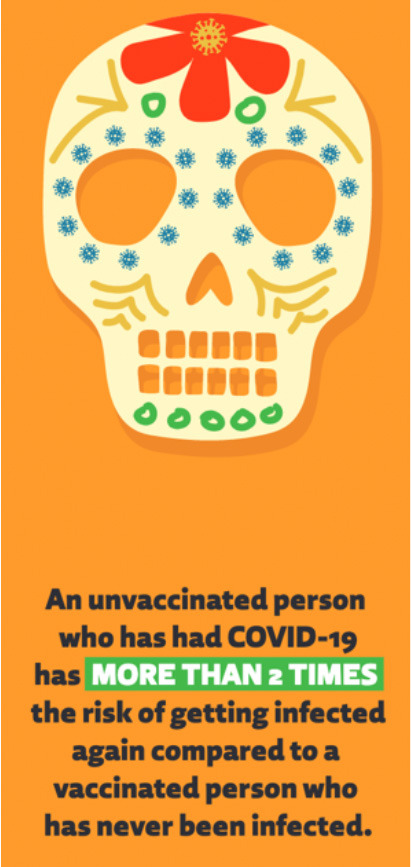
**Urban American Indian/Alaska Native**	•**Audience:** Adolescent (12–17 years old) •**Imagery:** Super Hero •**Tag line:** Getting vaccinated strengthens the whole tribe and community! •Did you know, coronaviruses have been studied for over 50 years! •Let's help life return to a new groove. •Fight the virus, bolt to your nearest location and get your free COVID−19 vaccine today •**Audience:** Older/elder (35–60+) •**Imagery/Tag line:** Life is sacred–Depicted with a Hummingbird •There are 3 highly safe and effective COVID−19 vaccines. •Keep our tribes safe and alive. •Heal our world, get your free COVID−19 vaccine today.	•In the fall of 2021, our workgroup focused on reaching two audiences: adolescents (12–17 years) and older adults/elders (35–60+ years) with two designs–a superhero and an elder. Recognizing the importance of matriarchal culture and appealing to the diversity of the Native community in Denver, both designs are women with Intertribal clothing and jewelry. The snowcapped mountains, Denver skyline, and Columbine flower were all carefully chosen to capture familiar, local images. •Rather than show needles or other images that may lead people to think about pain, the hummingbird is a symbol of hope, the superhero is powerful, and the older woman shows the importance of protecting elders.	•The group selected tangible products: masks, shirts, posters, stickers, backpacks, and postcards to distribute throughout the community. •Workgroup members printed t–shirts with the same images to wear as a uniform when rapid–CT group members distributed products. •Distribution plan focused on sharing products through schools, Native–serving organizations in the Denver metro area, and at events during the winter and spring (Denver March Pow–wow).	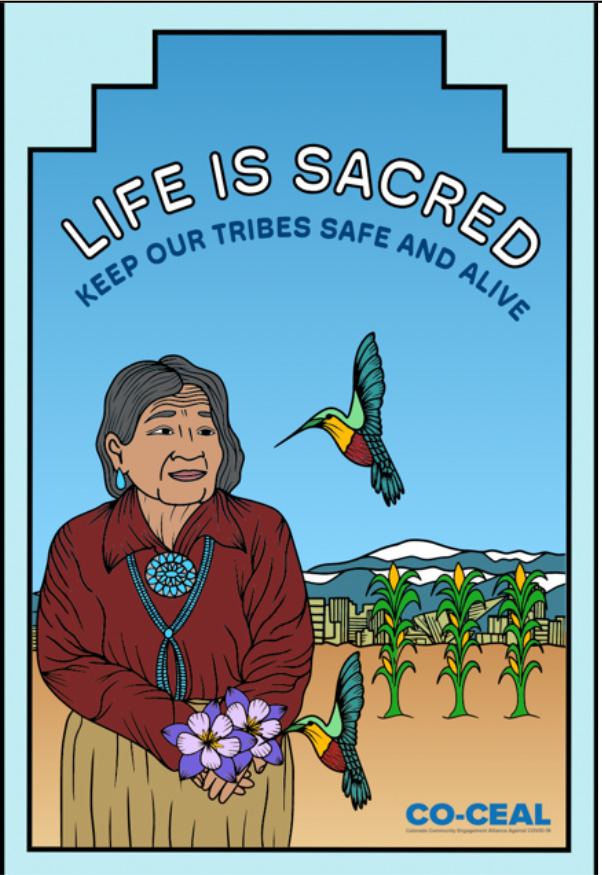
**Urban Black/African American**	Protect the Family: Get Vaccinated •Do it for us, do it for them, do it for you	•YouTube video produced by and featuring rapid–CT members •Website (thefam4vax.org) •Video and website focused on sharing the facts about the vaccine, including the involvement of the black community in its development •The group decided that a video that showing the family discussing COVID−19 during a holiday dinner. The importance of “Big Mama” (Mother/Grandmother) as the primary figure delivering the message was depicted; included a call to action for mothers and mother–figures to ask their families to get vaccinated	•Playing the video during actual family gatherings (during the 2021 holiday season) •Sharing on personal social media pages •Sharing with community and other gathering places, health care organizations that COVID−19 promote vaccines •YouTube Channel: https://youtu.be/zzvIOSajE2c	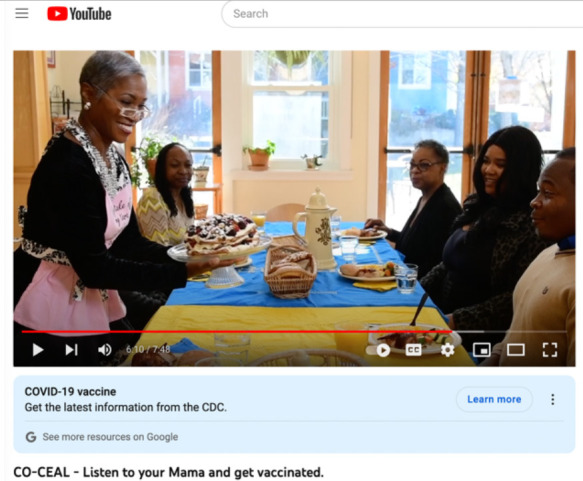
**Urban Latino/a/x**	•Together we can *(juntos si se puede)* •Vaccine is safe and easy to get •Cuidate tu para cuidarnos a nosotros. Mantenos seguros. Vacunate. #JuntosSiSePuede •Take care of you to take care of us. Keep us safe. Get the vaccine. #JuntosSiSePuede	•Spanish and English language bus ads and bench signs, posters and rack cards •Featured rapid–CT participants •Messages focused on care of/for yourself and others, save lives, hope, work together as a community •Safety and accessibility of vaccine •Vaccination protected community to just individual	•Bus ads and bench signs throughout community •Placed posters and rack cards in community areas, social media •Rack cards and masks distributed at local events	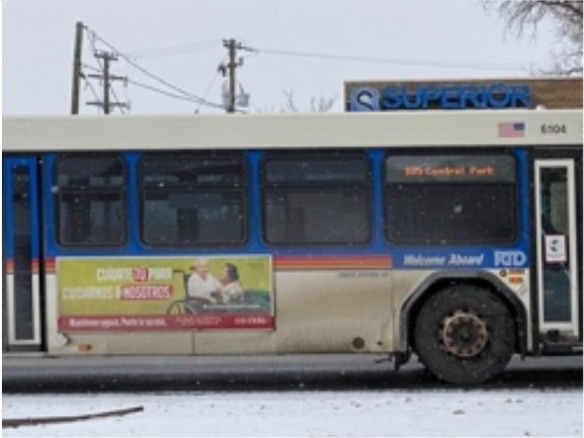

**Table 3b:** Cycle 2–Campaigns Promoting Child and Teen COVID−19 Vaccination.

Rapid–CT Team	Key messages	Description of campaign	Distribution strategies	Example product
**Rural African Immigrant**	•COVID−19 facts and effects on people of all ages, information on vaccines and how they help •Countering misinformation	•Participants created short video recordings about COVID−19 prevention and vaccine education in order to spread awareness in the community. Both male and female participants were selected to empower them to deliver this educational message with cultural responsiveness. •A large banner was printed for a local mosque •A colorful flier/tri–fold brochure was printed for distributing additional information.	•Banners hung at mosques, meetings and events for community members, canvasing local businesses and restaurants to hang flyers, sharing in breakrooms at work and the meat processing facilities. •Videos shared on social media	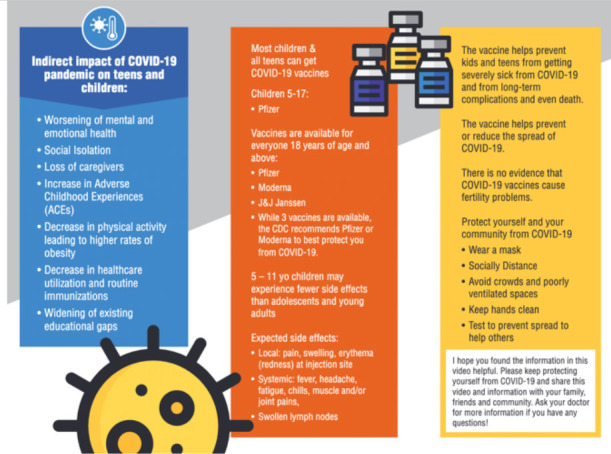
**Rural Latino/a/x**	•Focus on storytelling •There are multiple ways to be a superhero to keep yourself and others protected	•Spanish and English coloring book for kids with pages for parents, crayons, stickers •Coloring book described how to be a superhero around COVID	•School districts, including summer school programming •Boys and Girls Clubs, community gatherings, local clinics	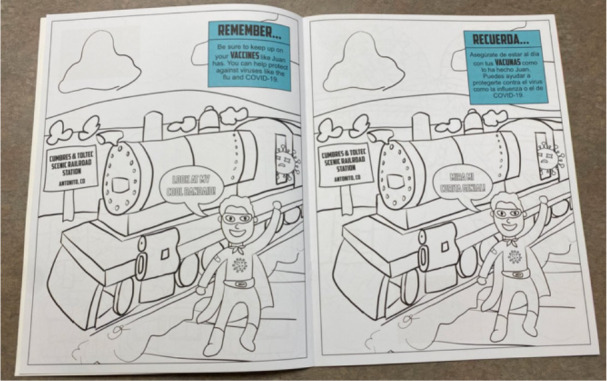
**Urban American Indian/Alaska Native**	•**Audience(s) for materials/messages:** 5 to 12 years old. Keep in mind who makes the decision is the caretakers. •**Tagline: Children are sacred. Protect your child today for their healthy tomorrow**. •Keep Are you aware that COVID−19 may lead to diabetes and other complications in children? •Your child can protect other children by getting a vaccine. •There is a safe and effective COVID−19 vaccine for our future generation. •Call to action: •**Let's keep our past, present**, and **future traditions alive** by getting **and get vaccinated**. •**Take the steps to get vaccinated!**	•The workgroup focused on creating an online ‘zine”„ a 10–page small–circulation self–published work. The group also printed water bottles, posters, stickers and t–shirts for children. •Used colors from American Indian Indigenous medicine quilt •Some of the key images were to capture intergenerational gatherings such as a talking circle with healthy food options. •**Footprints** to symbolize taking steps toward the future with different footprints representing tribes of the group members.	•Community and state–wide Pow Wows •The distribution approach was similar to Cycle 1 focusing on organizations, sporting events in the community and key events •In–person events and via social media	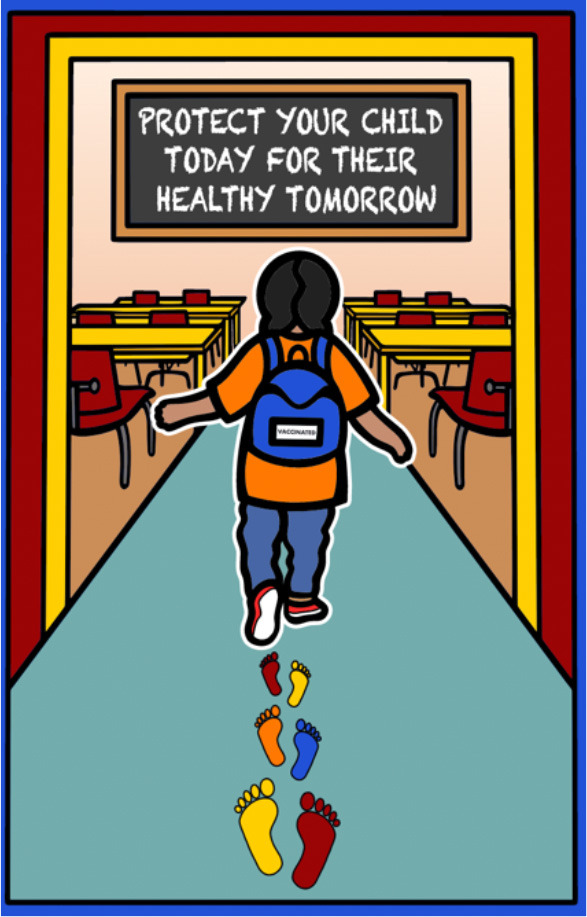
**Urban Black/African American**	•Keep your children safe, get them vaccinated! •Black children disproportionately experience more complications from COVID−19 infections. •Preventative measures, such as mRNA COVID−19 vaccines, mask wearing, hand washing, and healthy choices, keep your family safe. •The mRNA COVID−19 vaccine is safe and effective for children 5 years and older. •Diversify your research and information sources to make an informed decision for your family	•Identified parents and youth as key audience (1) parents are the ultimate decision–makers and need access to accurate information about the vaccine and (2) youth are being exposed to negative and false messaging about the vaccine •Counter false messages with messages and products that are positive and true and tap into the power of youth to influence one another and potentially their parents to get vaccinated. •Disseminate via both active and passive methods	•Distributed materials (flyers and small durables–backpacks, water bottles, grocery bags, stickers, notebooks, coffee mugs) to parents and youth at community events–church events, personal networks, backpack drives, Juneteenth community events •Bus bench/shelter signs in areas selected by the community with both high Black/African American populations and busy intersections	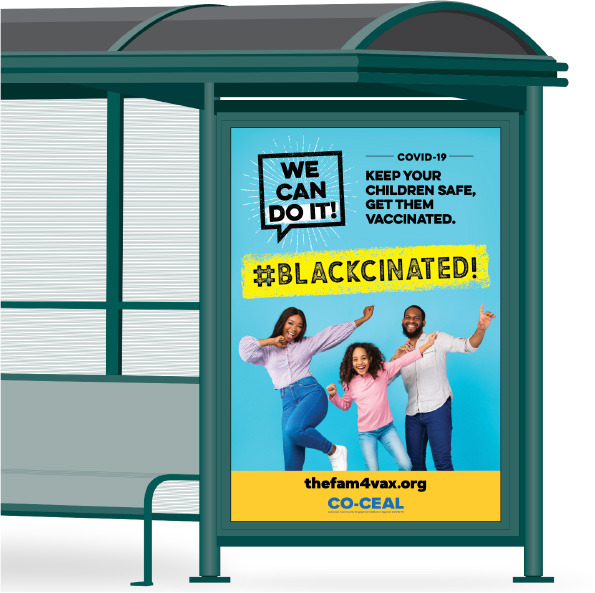
**Urban Latino/a/x**	•Get vaccinated •Protect your family and community	•Spanish and English language comic coloring book, crayons, stickers, website, social media •Coloring book featured two children getting vaccinated and becoming warriors with powers	•Social media •Coloring book contest on website, Instagram, Facebook •Coloring books shared at health and wellness events and other community events and organizations (state fair, Boys and Girls Clubs)	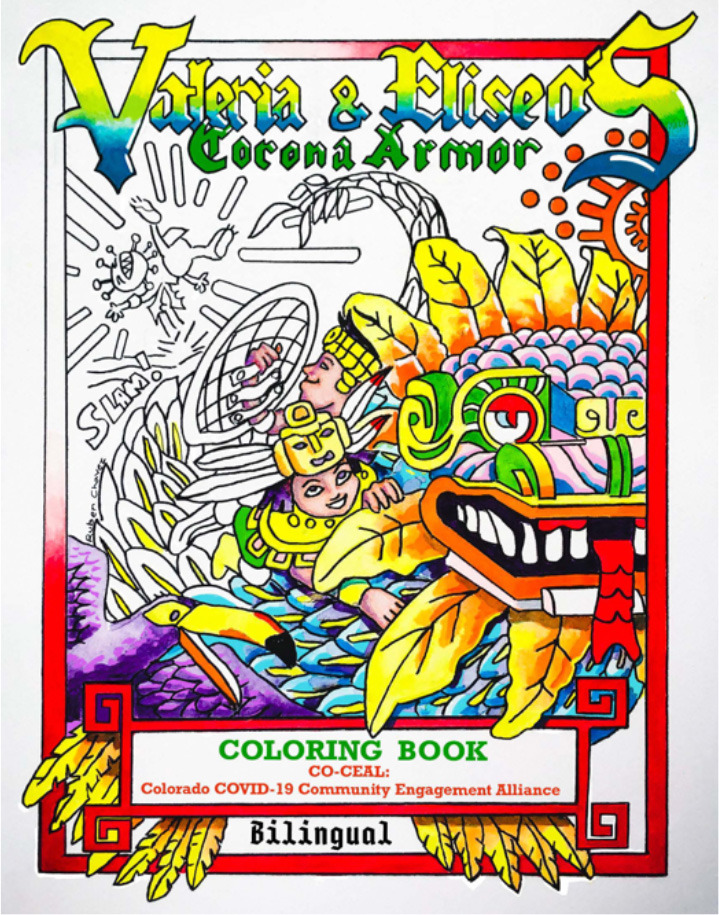

**Table 3c:** Cycle 2–Campaigns Promoting COVID−19 Vaccination and Long–COVID Prevention.

Rapid–CT Team	Key messages	Description of campaign	Distribution strategies	Example product
**Rural African Immigrant**	Focus on vaccination and safety (avoid risk of misinformation). These translated to: “COVID vaccine is important, please take it”; “the vaccine is important for your protection”; and “the booster is important for protection”	Intention was to “flood” the community with materials. Created 100 “kits” to go out to people; 50 kits per community. Kits included: pens, tote bags, water bottles, lunch cooler bags, key chain flashlights, keychains, glow in the dark wrist bands	Two community events, plus the tight–knit community shared materials and information with family and friends at mosques and businesses.	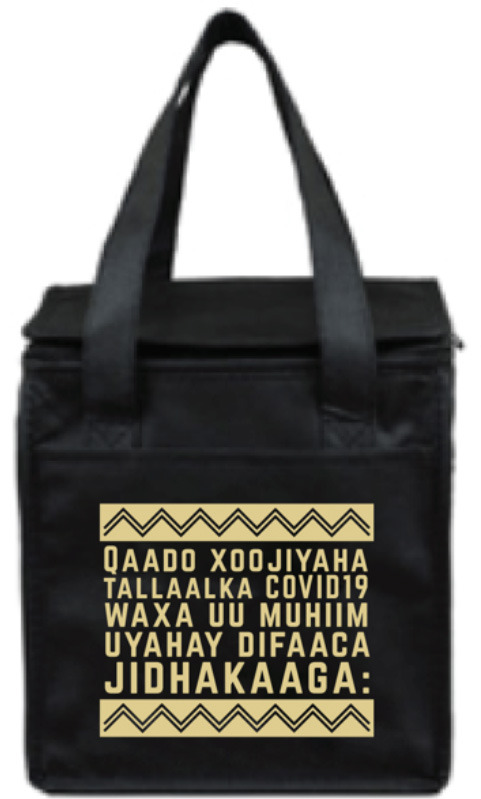
**Rural Latino/a/x**	Various COVID facts: •Being up to date with your covid vaccine & booster series reduces your risk of Long COVID. •COVID−19 infection in children increases their risk of Diabetes by 31% and by 40% in adults.	Idea is that book was to be completed by kids and their elders together. Kids and elders depicted as superheroes to fight illness. Focused on older adults and children, bivalent boosters, staying safe. activity book, included local and cultural references (e.g., marimba, church, labyrinth). Used English and Spanish language.	•Multilingual community health events with bring in the university, churches, schools •Posters for TikTok competition •Cinco de Mayo event •Missing and Murdered Indigenous Women's Pow Wow •Social media at local colleges •Local Public Health vaccine bus, Local Public Health Agencies and hospitals/primary care clinics •Daycare providers •School nurses	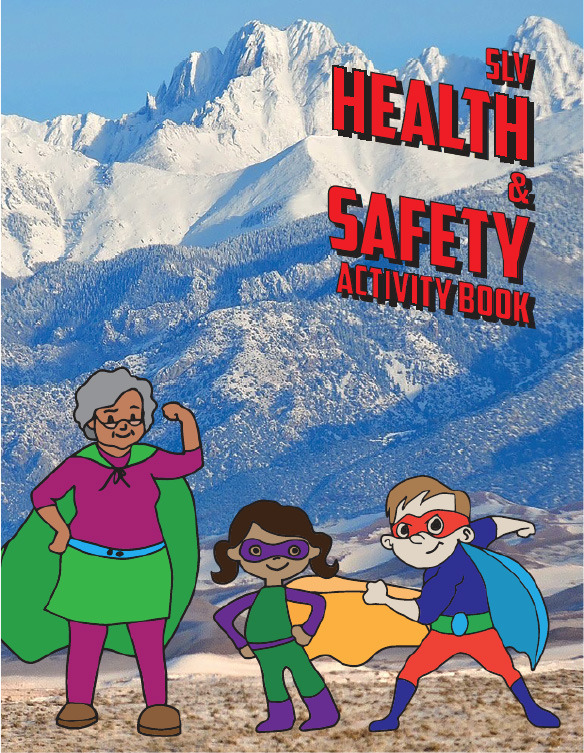
**Urban American Indian/Alaska Native**	“Share frybread, not COVID”; “Warriors against COVID”, “Spring forward fully vaccinated,” “The Effects of COVID−19 Vaccine Wane Overtime”	The community chose to have imagery of a more intentional gathering and to share frybread. Gatherings are so important and need to be safe. Other materials showed multigenerational images with language of helping protect each other. Additional language reminded people of the importance of boosters due to vaccine effectiveness waning over time.	•Aligned with the launch of COVID−19 bivalent boosters. •Local Powwow, •Local centers serving American Indian community members and patients •Schools: American Indian Academy and others serving public school students, after school programs •Indian Health Services Navajo Division Facebook page	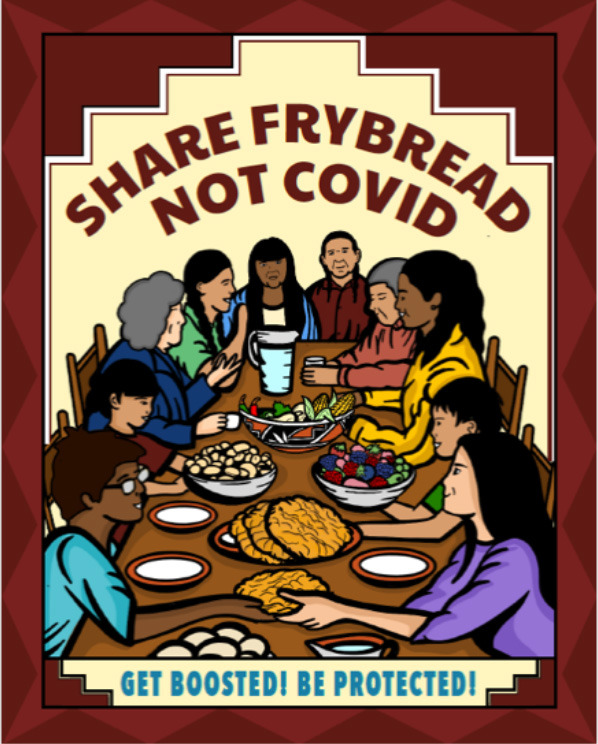
**Urban Black/African American**	•Protect the family •Covid is still dangerous and is a leading cause of death •20% of adult survivors report having long COVID and 38% of Black American with long COVID report significant limitations •Keep your family safe •Get vaccinated–Get boosted	The group created a variety of products to share with a list of select community locations to share with. Their materials included: flyers and posters fanny packs, grocery bags, journals, and fans, along with a brief movie trailer–style video and animations to encourage people to share online and through trusted social media channels.	Sent materials to a wide variety of community organizations	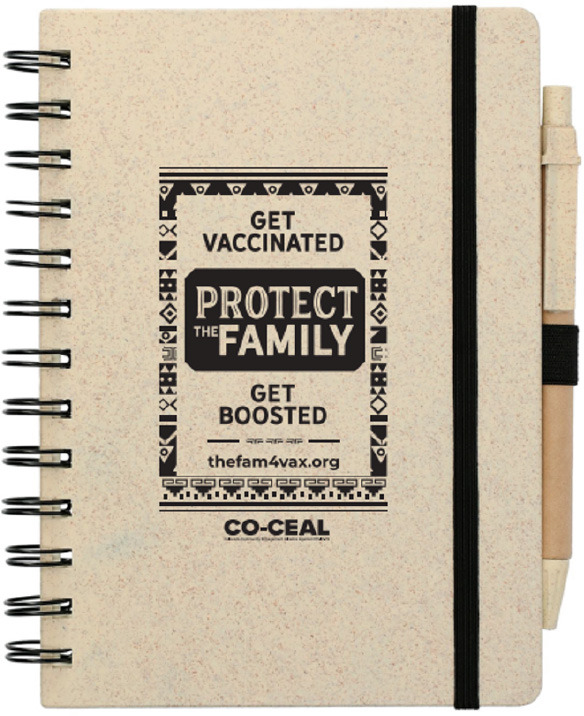
**Urban Latino/a/x**	The community wanted to be sure they emphasized the importance of finding vaccines and resources on COVID−19 and vaccines/boosters, and on educating the community around this as a priority.	Commissioned three pieces of art for dissemination materials. These images were printed onto calendars, flyers, and tote bags.	The flyers advertise the two planned community events: Cinco de Mayo and a family and community–friendly Safe Summer Kickoff. Community partners also assisted with distribution to various community locations.	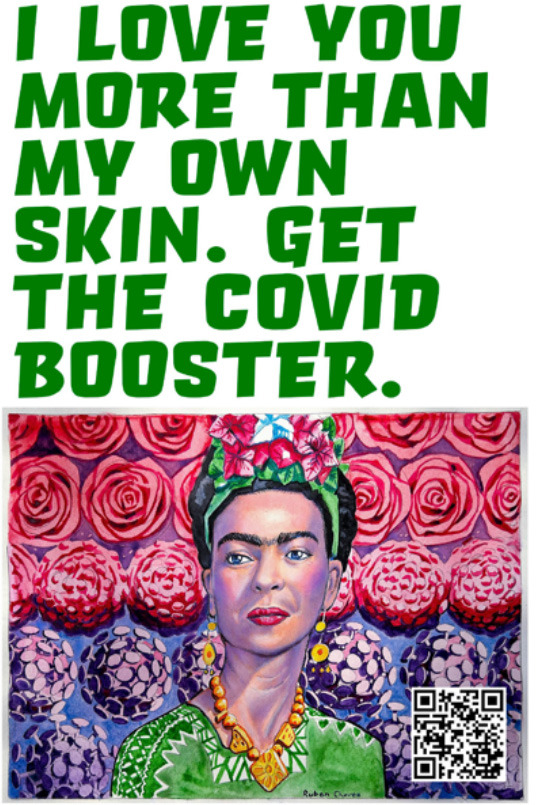

### Develop messages and materials describing COVID-19 vaccination, its rationale and evidence base, and its relevance to the communities of interest context (Step 2)

Key messages describing the COVID-19 vaccine and its importance for individual and population health were reported previously (40). Within each cycle, rapid-CT communities co-created distinctive campaigns including messages, materials, and dissemination strategies and plans, which are described in Tables 3a–3c.

### Describe the target audience for dissemination of COVID-19 vaccination messages and materials and the sequence, timing, and formatting of messages and materials (Step 3)

As part of the rapid-CT process, each rapid-CT team defined their target audience–usually by the third or fourth meeting. Most teams defined their audiences broadly given the urgent need to reach nearly all members of their pre-defined racial/ethnic and geographic communities. For example, the AI/AN team intentionally segmented the audience by age based on both who makes vaccine decisions (patient vs. Parent) and difference in guidelines by age. In cycle 2, a narrow focus was predefined on parents and/or caregivers of children between the ages of 5 and 17 years, given the project's overall focus on promoting COVID-19 vaccination for these age groups. In cycle 3, the audiences were largely generalizable since materials were targeting adults, youth, and those that were vaccine hesitant. Details of the target audiences defined by each rapid-CT team are presented in Tables 3a–3c.

### Define the communication channels for sharing COVID-19 vaccination messages and materials (Step 4)

The communication and distribution strategies for each rapid CT group are displayed in Tables 3a–3c.

### Barriers and facilitators of disseminating COVID-19 vaccination messages and materials in the intended communities (Step 5)

Each rapid-CT group identified anticipated barriers and facilitators in advance of dissemination, as well as tracking unexpected barriers and facilitators that emerged upon enacting the dissemination plans. Several anticipated barriers were common across communities, such as the widespread lack of trust in COVID-19 vaccination and public health messaging nationwide, which were also concerns in these communities. Community survey results indicated misinformation and lack of knowledge were ubiquitous (45). Other anticipated barriers included the rapidly changing data and information about COVID-19 and COVID-19 vaccines, the ability to receive the vaccine in their community, and language and cultural factors, which informed decisions about who would serve as medical experts and rapid-CT facilitators and use of interpreters. Barriers identified once dissemination of messages and materials were underway included timing messaging with COVID-19 evidence changes including that we are all “living within an endemic,” community events, as well as the communication with and availability/capacity of many of the trusted community messengers to distribute materials in community. It was not consistently made clear nor fully understood from the outset that this would be expected as part of the role of the engaged partners, or the scope of what this work would entail. For the communities where English was not the only spoken language, additional supports were needed to ensure products were both translated and interpreted into language that was not only accurate but also culturally- and community-specific. Some of the rapid-CT participants were confused about roles and responsibilities among the many members of the team. At times, there was misalignment between rapid-CT participants and facilitation team personality, organization, and style factors that made communication difficult. Confusion around product production processes and slow timelines for the dissemination materials caused issues in nearly all communities.

Facilitators common across communities included motivation to protect others within their family and community, such that more than half of survey respondents indicated wanting to protect themselves, their family, and others in their community by getting vaccinated (45). Additional supports that aided the process included the involvement of medical experts who were members of, or who spoke the language of, the focus communities, as well as groups that had at least a few returning rapid-CT member who could help guide the newer members through the process. Other factors included trust and support within the communities including clear and regular communication, the communities' understanding of resources in their community (e.g., designers, artists, and printers; upcoming events at which distribution of materials could occur), and relevant timelines and events (e.g., community, social, holidays, religious, etc.).

All of this impacts what the community sees and learns, but also impacts relationships, retention, and commitment.

### Evaluating the impact of the dissemination process. (Step 6)

The formal dissemination tracking spreadsheet used in cycle 1 was completed by one group. A revised tracking spreadsheet used for cycle 2 was more successful, with four communities at least partially completing the document. During cycle 3, the team chose a combination of the revised tracking spreadsheet and supplemented content learned through debrief and All Hands meetings to complete documentation. Additionally, the rapid-CT staff attempted a document review and determined there was no optimal way of quantifying the reach and impact in a way that both could be compared between communities and rapid-CT teams and could be aggregated to report on the project's impacts. What we found was that each community had a different “top metric” for community exposure to their products. These metrics of impressions could be reported depending on their product type and distribution channels (Table 4). For instance, in cycle 1, the Urban Black/AA group was able to track YouTube video views (400 views within 1 year). The Somali community's primary metric was number of community meetings held and number of attendees at each meeting (1 meeting; approximately 40 attendees). The Rural Latino group was able to track that they had four entries to their TikTok competition. The AI/AN group tracked number of materials distributed at the Denver March Pow Wow (distributed an estimated 150 tote bags, 100 mugs, 1,000 stickers, and 100 postcards). The Urban Latino group received metrics on reach of their bus ads and bench signs from the transportation district (in Pueblo, 4 bus benches estimated 93,000 weekly impressions based on geopath estimates). In cycle 2, the Urban Black/AA group tracked metrics for bus benches and signs (5 benches for 6 months, 50k views per week = 6 million traffic views (estimated)). The Rural Latino group tracked distribution of activity books (2,000 printed activity books). Additionally, survey data on exposure helped us assess the need for adaptations to the intended audiences (45).

**Table 4:** Top Metrics of dissemination by community and rapid–CT cycle.

Community dissemination by cycle	key metric and time frame
*Cycle 1 (adult COVID vaccines)*	
Urban Black/AA–YouTube video	400 views within first year/550 over a 2–year period; Additional untallied views at community organization and church–based discussion events
Somali–Somali/English Informational Flyers and Posters Community events	550 Copies distributed January/February 2022; 1 event on 1/23/2022 (estimated 40 attendees)
Rural Latino–TikTok competition (https://www.tiktok.com/tag/ourstoriesslvcovid) OurStoriesSLVcovid.com	4 entries; 2735 views (Contest winners); 472 Website views 1/1/2021–8/7/2023
AI/AN–Materials distributed at Denver Pow Wow, schools, American Indian–serving organizations: Masks (2 designs), stickers (2 designs), Posters (2 designs), postcards (2 versions) and backpacks.	100 masks; 2000 stickers; 30 posters; 250 backpacks; 17 tshirts; 200 postcards
Urban Latino–Bus ads and bench signs; Social media videos; Materials distributed at community events	Denver: 4–7 weeks of viewing time; Pueblo: 4 bus benches; est. 93,000 weekly impressions; Social Media: static images shared on the personal social media pages of participants; Rack Cards: 3,000; Masks: 300
***Cycle 2 (pediatric COVID vaccines)***	
Urban Black/AA–Bus benches	5 benches in all for 6 months, 50k views per week = 6 million traffic views (estimated)
Somali–Somali/English Materials shared at community events/delivered to local businesses, restaurants, and mosques Videos shared on WhatsApp	5 events, 6 businesses (500 brochures printed); “Majority of people” in 2 cities
Rural Latino–Coloring books distributed to schools, clubs, WIC offices, vaccine buses	3,386 coloring books distributed
AI/AIN–Focus on school based organizations/cubs Pocketbook sized ‘zine, posters, water bottles, t–and shirts for youth and adults	1,000 paper copies and online flip book30 taking steps poster; 50 elder talking circle poster; 400 water bottles; 110 youth t–shirts; 24 adult t–shirts
Urban Latino–Comic Book (in English and Spanish)	4,000 total printed (2,000 per community location)
***Cycle 3 (Long COVID, COVID**−**19 Boosters, “Late Adopters”)***	
Urban Black/AA–Flyers and posters; Fanny packs, grocery bags, journals, fans; Movie trailer and animations	1100 copies printed/distributed; 1725 produced/distributed; Trailer: 10 trackable views^*^
Somali–Community informational kits—tote bag filled with cooler, pen, water bottle, flashlight, keychain, glow wrist band	100 total (50 kits per community location)
Rural Latino–Activity book; TikTok contest	1,000 activity books; 2 TikTok submissions
AI/AIN–Tote bags and ceramic mugs	300 tote bags; 216 ceramic mugs
Urban Latino−3 commissioned pieces of art: printed on grocery bags, flyers, and calendars	1,000 grocery bags; 1,000 flyers; 200 calendars

^*^The group utilized a grass roots approach in which all rapid-CT group members had the original files and uploaded and posted them to their own networks, so not all views were track-able by the CO-CEAL central team.

## Discussion

CO-CEAL engaged communities in design and dissemination of products using 3 cycles of the rapid-CT method to promote COVID-19 vaccination in 5 underserved and marginalized communities in Colorado. Using Bauman et al.'s dissemination planning framework (41, 42) to describe and evaluate this work, rapid-CT was effective for engaging communities in co-creating distinct messages and communication strategies tailored to their community needs and subsets of the population who most needed to receive that messaging. Communities selected a wide range of local messengers and communication and distribution channels for reaching their communities, ranging from common marketing approaches (e.g., bus ads) to social media (e.g., TikTok) to community events (e.g., Denver Pow Wow). While tracking impact of dissemination was challenging, ultimately an idiosyncratic approach based on community-specific capacities and product types was useful to demonstrating reach to intended audiences.

Our application of the Rapid-CT method to increase COVID-19 vaccination uptake in communities revealed similarities and differences compared to traditional Boot Camp Translation. First, the communities similarly were able to translate complex medical evidence to their community culture and context but in a shorter amount of time, 6–8 weeks compared to 6–9 months. For example, the Testing to Prevent Colon Cancer project created a suite of products to promote colon cancer screening in rural Colorado that are uniquely tailored to rural farm and ranch cultures and tracked reach and exposure to those materials in intervention and control counties as part of their outcome analysis (27, 46). Similarly, the ITMATTTRS study tracked reach of BCT-created materials by products printed and distributed in their communities (30). Both these examples of projects using BCT were targeting diseases and health behaviors with more stable information, incidence, and treatment options in communities. These factors were key barriers to tracking and evaluation in the CO-CEAL project in which COVID-19 information, community incidence, and available treatment options (both vaccination and medication) changed multiple times during the course of the project. This suggests both that rapid-CT is better suited to message creation for dynamic public health emergencies.

A challenge in use of rapid-CT related to the community partner time commitment that was difficult to predict in advance and in the context of an evolving pandemics impacts on participants. Rapid-CT work goes beyond 8 weekly meetings; it also includes enacting the dissemination plans and tracking and evaluating the impact. Given the dissemination plans were not known at the outset when agreements were made with community partners, there was wide variability in the time and intensity of this work. This made it difficult to appropriately set expectations of the commitment for rapid-CT participants in advance. Future applications of the rapid-CT process could address this by more clearly communicating the expectation to be engaged in tasks between meetings, dissemination and tracking after the 8-week series of meetings, and facilitating the creation of the dissemination plan such that tasks are clearly assigned to each individual involved. Better understanding and acknowledging who has the capacity, expertise, and resources (community and academic partners and personnel), especially with respect to enacting the dissemination plans and tracking outcomes, is needed.

Despite using a rapid version of the message development process, the pace still felt too slow for some involved given the quickly evolving evidence on COVID-19 vaccination, as well as changing recommendations on vaccination during this period. As science was changing on a weekly or monthly basis, presentations from a medical expert leading up to a rapid-CT event had to be updated and data were sometimes out-of-date before key messages were selected by the community members. This required more than average time and communication with the medical experts after kick-off meetings. That said, groups with more consistent medical expert support felt more confident in the data presented in their final materials. Our rapid-CT teams had to navigate the constantly changing landscape of evidence and adjust their messaging strategies accordingly.

### Limitations

Despite the successes of this approach, the rapid-CT process was not without its limitations. While the rapid-CT process is well-suited to quick translation of medical evidence, the rapidly evolving data that emerged during the process created challenges for developing messages and materials. For example, recommendations about who should get the vaccine evolved. When cycle 2 began, vaccines were approved for children over 5 years; just weeks after materials were printed vaccines were approved for children over 6 months. Current events (e.g., Nicky Minaj's tweet about the vaccine and infertility; Colin Powell's death, and involvement of Dr. Kizzmekia Corbett (a Black Research Scientist who worked on the development of the COVID-19 vaccine)) also shifted team conversations about COVID-19 vaccines and influenced message creation. During support meetings, some facilitators reported challenges around the pace of the rapid-CT. However, all understood the urgency of the situation and completed the cycles successfully.

Additionally, within communities there was great heterogeneity in vaccine acceptance, belief about the vaccines, how messages and information are processed, etc. We attempted to capture this through diversity in rapid-CT team make-up; however, there was likely bias in who was willing to participate in a process to create materials focused on promoting COVID-19 vaccines. Multiple teams included participants who were hesitant or did not vaccine themselves or their children. Nonetheless, they engaged fully in the process and in disseminating final materials in the community, suggesting the process was engaging and educational regardless of personal beliefs.

Finally, tracking and evaluation of impact of rapid-CT messaging involved significant challenges with respect to standardization and availability of data to assess reach and influence on vaccine behavior. It was difficult to standardize dissemination tracking across the communities–things moved quickly, each group tracked their group's process differently, tracking ended up being mostly done retrospectively rather than prospectively. Additionally, due to the open-ended nature of the rapid-CT process, there were few standardized metrics established for tracking early on. The simple, community-specific tracking processes and metrics helped ensure community involvement in evaluation and mitigate the challenges with tracking. Future iterations will require more pre-planning to prospectively track reach and evaluate impact using common standardized metrics in addition to those defined by rapid-CT group members.

## Conclusions

The rapid-CT process enabled our CO-CEAL teams to translate and disseminate evidence regarding COVID-19, vaccines and Long COVID into community specific, relevant messages and materials across five diverse communities. While challenges regarding evolving evidence, diverse dissemination capacities and the overall rapid nature of the process were present, the overall goals of disseminating factual, relevant information into disproportionally impacted communities were met. Use of rapid-CT should be considered for addressing similar needs for rapid information transcreation during future health emergencies, while incorporating lessons learned regarding dissemination capacities and challenges.

## Data Availability

The raw data supporting the conclusions of this article will be made available by the authors, without undue reservation.
